# Can Fear of COVID-19 Be Predicted by Religiosity and Trust in Institutions among Young Adults? A Prospective Cross-National Study

**DOI:** 10.3390/ijerph19116766

**Published:** 2022-06-01

**Authors:** Dominika Ochnik, Aleksandra M. Rogowska, Ana Arzenšek, Joy Benatov

**Affiliations:** 1 Faculty of Medicine, University of Technology, 40-555 Katowice, Poland; 2 Institute of Psychology, University of Opole, 45-052 Opole, Poland; arogowska@uni.opole.pl; 3 Faculty of Management, University of Primorska, 6101 Koper, Slovenia; ana.arzensek@fm-kp.si; 4 Department of Special Education, University of Haifa, Haifa 3498838, Israel; jbentov2@gmail.com

**Keywords:** fear of COVID-19, religiosity, trust in institutions, young adults, a longitudinal study design

## Abstract

The aim of this study was to reveal whether religiosity and trust in institutions are longitudinal predictors of change in fear of COVID-19 (FCV-19) across Poland, Germany, Slovenia, and Israel among young adults over a three-month period. The representative sample consisted of 1723 participants between the ages of 20 and 40 years (*M* = 30.74, *SD* = 5.74) across Poland (*n* = 446), Germany (*n* = 418), Slovenia (*n* = 431), and Israel (*n* = 428). The first measurement was carried out in February 2020 and the second was conducted in May/June 2020. A repeated-measures, two-way, mixed-factor ANOVA was performed to examine changes over time (T) and across countries (C) as well as the interaction of time and country (TxC) for FCV-19, religiosity, and trust in institutions. The results showed a significant decrease over time and differences between countries in all variables, as well as in TxC for FCV-19 and trust in institutions. Linear generalized estimating equations (GEEs) were used to assess the longitudinal change between T1 and T2 in FCV-19, including religiosity and trust in institutions as predictors, country as a factor, and gender and age as confounders. Female gender, religiosity, and trust in institutions were found to be significant longitudinal predictors of change in FCV-19. Country was a significant moderator of the relationship between trust in institutions and FCV-19, with the highest result achieved in Poland. Religiosity and trust in institutions were positive predictors of change in fear of COVID-19 among young adults across countries. Religious and governmental institutions should take this into consideration when communicating with believers and citizens during challenging situations.

## 1. Introduction

The coronavirus disease 2019 (COVID-19) pandemic disrupted everyday life globally due to restrictions regarding social distancing, cancellation of public events, and hardships in traveling and commuting [1,2]. The pandemic affected not only the physical health but also the mental health of the population worldwide [3]. To prevent the spread of the virus, government officials imposed unprecedented measures such as closing down public and private facilities and enforcing social distancing [2]. Insecurities about health and feelings of isolation led to increased levels of stress, depression, and anxiety [4,5,6]. Religious institutions were also affected by the lockdown, which led to a lack of social and emotional support in religious communities [7].

Religiosity can serve as an anchor for many people during times of crisis [8,9]. Religious beliefs provide a sense of security and reduce fear and anxiety [10]. There are associations between religiosity and mental health; however, the findings have been mixed. Some studies have reported positive relationships between religiosity and mental health and have concluded that religiosity can buffer the negative consequences of psychological distress [11,12,13]. For instance, religiosity is associated with increased psychological well-being and life satisfaction [14,15,16] and lower levels of depression, anxiety, and distress [15,17,18]. During the COVID-19 pandemic, several studies found that religiosity helped people to deal with stressful life events [18,19,20,21,22]. In contrast, other studies have not found significant associations between religiosity and mental health outcomes [23,24] or have found religiosity to be a small but significant predictor of higher stress levels during the COVID-19 pandemic [25,26]. Early meta-analyses reported small-to-moderate positive relationships between religiosity and mental health [27,28,29,30]. However, more recent meta-analyses, including longitudinal studies, have only found small associations [31,32].

Furthermore, trust in institutions could have been disrupted by the pandemic [33]. Trust in institutions is crucial during a crisis as it elevates the government’s capacity to pursue redistributed polices [34] and encourages adherence to health policies [35]. Previous research has shown that trust in political authorities increased after outbreaks [36], and lockdown increased trust in the government in Europe [37]. However, panel data from the Netherlands did not confirm this association [38]. Other research [39] has shown that, in Spain, persons with direct exposure to COVID-19 expressed lower levels of trust. Furthermore, cross-national research has shown that risk perceptions regarding COVID-19 are lower when trust in the government is higher [40]. Trust in institutions can be affected by evaluations of how the pandemic is managed, as shown by the previous N1H1 pandemic [41]. Research conducted in 25 European countries has revealed that trust in institutions is also positively related to a lower mortality rate [42].

The relationships between religion and social trust as a wider notion, compared to trust in institutions, have been neglected thus far [43]. However, in previous research, the results have been mixed. Some research has found no significant relationship between religiosity and social trust [44]. Other research has shown that even though there is clear association between practicing religion and trust, an affiliation with Christianity is related to lower trust [45], while among Latin Americans, a Christian affiliation is positively related to trust [46]. Furthermore, religiosity has been shown to be negatively related to trust in institutions (politics and “good government”) [47].

Fear of COVID-19 is a key mental health index related to the COVID-19 pandemic [48]. The prevalence of fear of COVID-19 ranges between 18.1% and 45.2% [49]. Younger adults, women, urban residents, divorcees, healthcare workers, people in quarantine, those with a higher risk of being infected, and those with mental health problems are at particularly high risk. The changes in fear of COVID-19 during the pandemic situation were large, while other metal health indices, such as perceived stress, anxiety, and depression, were small or insignificant [50,51]. Moreover, the fear of COVID-19 intensity fluctuated along with the mortality rate [51].

Despite the growing literature related to the COVID-19 pandemic, many aspects are still unclear. In this study, we investigated the role of religiosity and trust in institutions in the fear of COVID-19 from a cross-national and prospective perspective among representative samples of young adults from Poland, Germany, Slovenia, and Israel. These countries represent the cultural diversity of traditional vs. secular and survival vs. self-expression values based on the Inglehart–Welzel World Cultural Map [52]. The two dimensions of values aggregate countries into clusters. Poland and Slovenia represent Catholic Europe, while Germany represents Protestant Europe, and Israel—West&South Asia. Among those countries, the most traditional values were found in Poland, while the greatest self-expression values were found in Germany. Poland, Israel, and Slovenia share similar values of self-expression, while Germany and Slovenia share similar secular values (to a higher degree than Israel). Furthermore, the countries vary regarding levels of trust in institutions, with Germany and Israel representing higher trust, while Poland and Slovenia showed lower trust [53].

Considering the relationship between religiosity and mental health issues [8,9,10,11,12,13,14,15,16,17,18,19,20,21,22,23,24,25,26,27,28,29,30,31,32] and trust in institutions [33,34,35,36,37,38,39,40,41,42] during the COVID-19 pandemic, we expected that these variables would be predictors of change in fear of COVID-19 over a three-month period during the change in the pandemic situation. The number of total vaccinations visibly increased while the stringency of restrictions and mortality rate due to COVID-19 decreased over the three months in each country [50,54]. We expected that these changes would be particularly significant among young adults at high risk of mental health deterioration during the pandemic [49,50], but would differ between countries [52,53].

Therefore, the aim of this study was to reveal differences in fear of COVID-19, religiosity, and trust in institutions across four countries over a three-month period. Furthermore, we aimed to show the role of religiosity and trust in institutions as longitudinal predictors for change in fear of COVID-19 with regard to country. We proposed a prospective cross-national study design to explore the fear of COVID-19 during the pandemic. 

## 2. Materials and Methods

### 2.1. Study Design

The present study adopted a longitudinal design among representative samples of young adults from Poland, Germany, Slovenia, and Israel. The data were collected by the ARIADNA panel over a three-month period. The first measurement (T1) was conducted between 19 and 26 February 2021, and the second measurement (T2) was taken between 26 May and 9 June 2021. The inclusion criteria were age between 20 and 40 years and country. To address potential sources of bias, the samples were representative in terms of gender, student status, and employment status. The participants were enrolled in a reward system (points exchanged for prizes, cash, or charity donations).

The survey was prepared in the native language of each country. The survey questions were translated from English by translation experts from the four countries according to the cross-cultural adaptation standards [55]. The study was conducted online. The participants answered all questions, as responses were required to continue the survey. There was no time limit. Furthermore, the participants could stop at any moment and return to finish the survey. The average time taken to complete the survey was 21.52 min (*SD* = 136.75). 

There were 2951 participants in the first study measurement (T1). Nevertheless, during the second measurement (T2), 1227 respondents failed to participate. Therefore, the research group comprised 1724 respondents who participated in both T1 and T2. The response rate was 58.42% in T2. One observation was excluded from T2 due to anomaly detection. Hence, the final total sample consisted of 1723 participants from Poland, Germany, Slovenia, and Israel.

This paper forms part of the international project “Mental health of young adults during the COVID-19 pandemic in Poland, Germany, Slovenia, and Israel: A longitudinal study” [56].

### 2.2. Participants

A representative sample of 1723 adults from Poland (*n* = 446; 26%), Slovenia (*n* = 431; 25%), Israel (*n* = 428; 25%), and Germany (*n* = 418; 24%) participated in the study. The mean age of the participants was 31 years (ranging between 20 and 40 years; *M* = 30.74, *SD* = 5.74). Women constituted 54% (*n* = 935) of the total study sample. A total of 49% of the participants were younger adults (*n* = 840) aged between 20 and 30 years. The majority of participants were employed (77%; *n* = 1324), coupled (71%; *n* = 1218), child-free (58%; *n* = 1001), and living in a town or city (75%; *n* = 1297). Detailed sociodemographic characteristics are presented in Table 1. 

### 2.3. Measurements

#### 2.3.1. Fear of COVID-19

The Fear of COVID-19 Scale (FCV-19S) evaluates fear of COVID-19 [48]. The FCV-19S utilizes a five-point Likert-type scale (from 1 = strongly disagree to 5 = strongly agree) and consists of seven items. The total score ranges from 7 to 35; the higher the score, the greater the fear of COVID-19. Cronbach’s α for FCV-19 was 0.91 at T1 and 0.92 at T2 in this study.

#### 2.3.2. Trust in Institutions

The Trust in Institution Scale is a part of social capital based on The European Social Survey [53]. It consists of three items relating to trust in parliament, trust in the legal system, and trust in politicians, evaluated on an 11-point scale, ranging from 0 = no trust at all to 10 = complete trust. The higher the score, the higher the trust in institutions. Cronbach’s α for the trust in institutions scale was 0.88 at T1 and 0.87 at T2 in this study.

#### 2.3.3. Religiosity

Self-reported religiosity was measured as an answer to the question “How religious do you consider yourself to be?” on a four-point Likert-type scale (from 0 = not at all religious to 3 = very religious). The variable was based on the Baylor Religion Survey [57].

#### 2.3.4. Religion

The question regarding religion was based on the Baylor Religion Survey [57]. The participants were asked to mark the one religious group, if any, that they most closely identified with. The possible answers were: No religion; Buddhist; Catholic/Roman Catholic; Hindu; Jehovah’s Witness; Jewish; Methodist; Muslim; Orthodox (Eastern, Russian, Greek); Protestant; other. 

#### 2.3.5. Sociodemographic Data

The sociodemographic data included gender, age (20–30 or 31–40 years), place of residence (village, town, or city), employment status (employed or unemployed), relationship status (single or otherwise), and having children (with children or child-free). 

### 2.4. Statistical Analyses

The fear of COVID-19, religiosity, and trust in institutions showed good psychometric properties at both timepoints, T1 and T2, during the pandemic. The variables conformed to the fundamental premises of parametric tests regarding the homogeneity of variance. The Kolmogorov–Smirnov test with Lilliefors significance correction and the Shapiro–Wilk test were conducted with regard to normal distribution; even though the analysis did not prove a normal distribution of variables (*p* < 0.05), further analysis of the distribution based on skewness and kurtosis coefficients indicated good symmetry and similarity to the Gaussian curve, because the absolute values of skewness did not exceed 1, which indicates good psychometric properties [58]. However, kurtosis for religiosity slightly exceeded 1. Nevertheless, these values for kurtosis are also considered to acceptably represent a normal distribution [59]. Hence, parametric analyses were introduced. 

Religiosity, trust in institutions, and fear of COVID-19 were continuous variables, while gender (women or men) and age (younger adults aged between 20 and 30 years or older adults aged between 31 and 40 years) were the categorical variables. 

The first step tested between-group differences in the 2 (Time: T1 and T2) × 4 (country: Poland, Germany, Slovenia, or Israel) repeated-measures, two-way analysis of variance (ANOVA). Effect size was estimated using the η² coefficient (small effect if η^2^ > 0.01, medium for η^2^ > 0.06, and large when η^2^ > 0.14) [60]. Tukey’s honest significant difference (HSD) test was conducted to examine the post-hoc group’s means comparison. The effect size for the post-hoc test was estimated with Cohen’s *d* coefficient (small for *d* = 0.20, medium when *d* = 0.50, and large if *d* = 0.80) [60]. 

The second step was to examine the associations between variables. Pearson’s correlation was performed as a preliminary analysis to find relationships in the total sample. Next, linear generalized estimating equations (GEEs)with robust standard error and an independent working correlation structure were used in the study to assess the longitudinal change between T1 and T2 in fear of COVID-19 (as a dependent variable), religiosity and trust in institutions as predictor variables, country (coded: Poland = 1, Germany = 2, Slovenia = 3, and Israel = 4) as a factor, and gender (men = 0 and women = 1) and age (older adults = 0 (aged between 30 and 40 years) and younger adults = 1 (aged between 20 and 29 years)) were included in the regression model as confounders. As the analysis requires a distinction between predictor and outcome variables, each model used the change in mental health as an outcome variable. 

**Figure 1:**
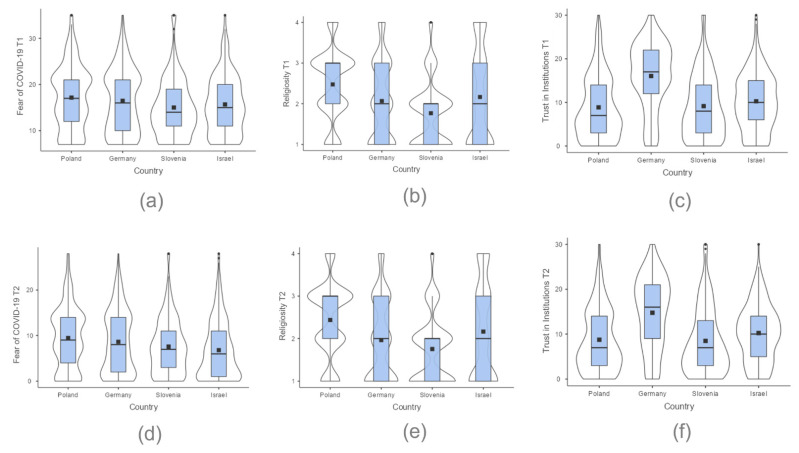
Distribution and mean scores of: (**a**) Fear of COVID-19 at T1; (**b**) religiosity at T1; (**c**) trust in institutions at T1; (**d**) fear of COVID-19 at T2; (**e**) religiosity at T2; (**f**) trust in institutions at T2 across Poland (PL), Germany (GER), Slovenia (SL), and Israel (ISR) among representative samples of young adults (*N* = 1723) shown by violin and box plots. Mean scores are represented by squares and outliers by dots. T1, the first measurement in February 2021; T2, the second measurement in May–June 2021.

**Figure 2:**
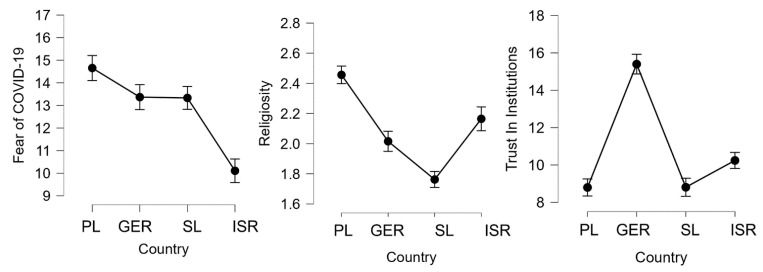
Fear of COVID-19, religiosity, and trust in institutions across Poland (PL), Germany (GER), Slovenia (SL), and Israel (ISR) among young adults (*N* = 1723) with error bars.

**Figure 3:**
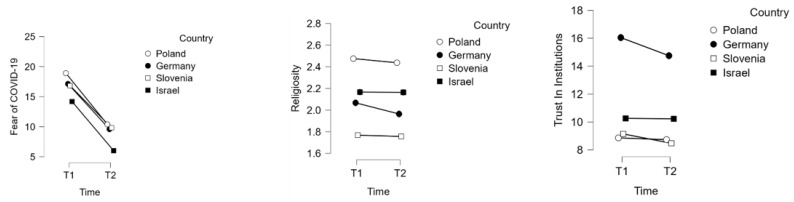
Changes in fear of COVID-19, religiosity, and trust in institutions over a three-month period across countries among young adults (*N* = 1723). T1, the first measurement in February 2021; T2, the second measurement in May–June 2021.

**Figure 4:**
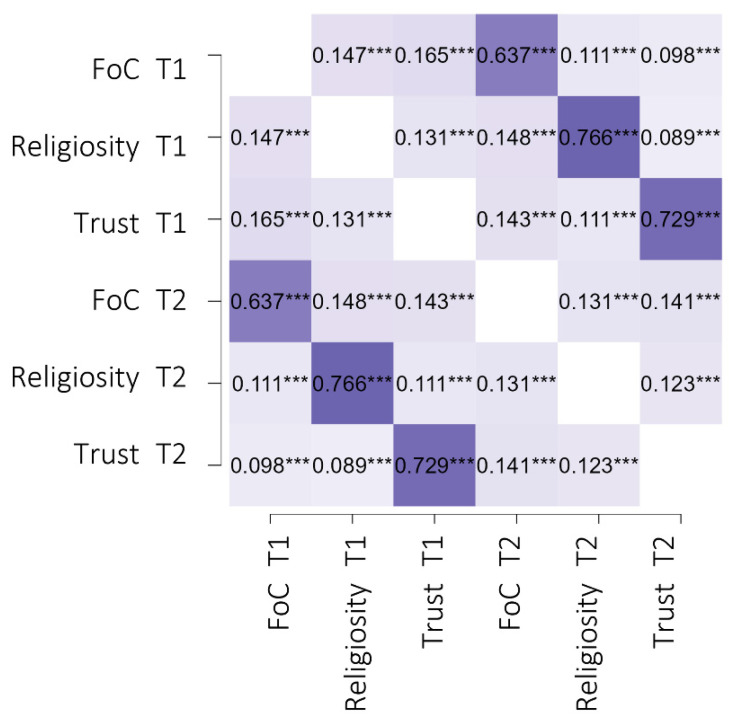
Heat map of the correlational matrix for fear of COVID-19 (FoC), religiosity, and trust in institutions (Trust) at T1 and T2 among young adults (*N* = 1723). T1, the first measurement in February 2021; T2, the second measurement in May–June 2021. Light violet indicates a small effect size; dark violet represents a large effect size. *** *p* < 0.001.

The statistical analyses were performed using JASP Team [61], except for the GEE analysis, which was performed in IBM SPSS Statistics 26 [62]. Figure 1 was created in Jamovi [63] and Figures 2–4 in JASP [60]. G*Power [64] was used to calculate the appropriate sample size. For the repeated-measures, two-way, mixed-factor ANOVA, the expected sample size was 158, assuming the two groups and two measurements, effect size *f*^2^ = 0.25, repeated measures *r* = 0.50, *p* < 0.05, and 95% *CI*.

## 3. Results

### 3.1. Differences in Fear of COVID-19, Religiosity, and Trust in Institutions across Countries

**Hypothesis** **1** **(H1).** *There are significant differences in fear of COVID-19, religiosity, and trust in institutions across Poland, Germany, Slovenia, and Israel over a three-month period*.

Due to objective changes in the COVID-19 pandemic situation in Poland, Germany, Slovenia, and Israel [50,54], we assumed that fear of COVID-19, religiosity, and trust in institutions would differ in T2 compared to T1. Furthermore, we hypothesized that there would be differences across countries attributable to cross-cultural differences. In the first step, we provided descriptive statistics, including distribution and mean scores for fear of COVID-19, religiosity, and trust in institutions in each country at two measurement points (T1 and T2). The details are presented in Figure 1. 

The second step involved a repeated-measures, two-way, mixed-factor ANOVA for fear of COVID-19, religiosity, and trust in institutions across countries (country: Poland, Germany, Slovenia, and Israel) over a three-month period (T1 = February 2021 and T2 = May–June 2021). We showed within-subject effects for time (T) and the interaction between time and country (TxC). We also revealed a between-subjects effect for country (C). 

H1 was confirmed. There was a significant difference in fear of COVID-19, religiosity, and trust in institutions between T1 and T2. Fear of COVID-19, religiosity, and trust in institutions significantly dropped over the three-month period. The effect size was medium for fear of COVID-19 and very small for religiosity and trust in institutions. The effects were significant across countries with a medium effect size for fear of COVID-19 and religiosity. The effect size for trust in institutions across countries was large. The interaction between time and country was significant for fear of COVID-19 and trust in institutions with a small effect size. However, the interaction was insignificant for religiosity across countries and time. The detailed statistics for fear of COVID-19, religiosity, and trust in institutions across countries are presented in Table 2.

Furthermore, post-hoc analysis with Tukey’s HSD test showed significant mean differences for fear of COVID-19, with the highest score found in Poland and the lowest in Israel. FCV-19 in Poland was slightly higher compared to in Germany (*d* = 0.18, *p* = 0.015) and Slovenia (*d* = 0.19, *p* = 0.015). Young adults in Poland reported significantly higher scores compared to their peers in Israel, with a medium effect size (*d* = 0.65, *p* < 0.001). The scores in Germany and Slovenia were similar (*p* > 0.05). However, Slovenia scored higher compared to Germany, but the effect size was small (*d* = 0.46, *p* < 0.001). 

Tukey’s HSD test for religiosity showed that, in Poland, religiosity was significantly higher compared to in all other countries. A small effect size was observed in comparison to Germany (*d* = 0.45, *p* < 0.001) and Israel (*d* = 0.30, *p* < 0.001), while a large effect size was seen for Slovenia (*d* = 0.71, *p* < 0.001). In Germany, religiosity was slightly higher than in Slovenia (*d* = 0.26, *p* < 0.001), while in Slovenia, it was slightly lower than in Israel (*d* = 0.41, *p* < 0.001). There were no significant differences in mean between religiosity in Germany and Israel. 

Trust in institutions was significantly higher in Germany compared to Poland (*d* = 0.93, *p* < 0.001), Slovenia (*d* = 0.93, *p* < 0.001), and Israel (*d* = 0.37, *p* < 0.001). The effect size for all countries was large. Trust in institutions was higher in Israel compared to Poland (*d* = 0.20, *p* = 0.006) and Slovenia (*d* = 0.20, *p* = 0.007), although the effect size was small. Similar mean scores were noted in Poland and Slovenia (*p* > 0.05). Therefore, the highest trust in institutions was reported in Germany and the lowest in Poland and Slovenia. The details for between-subjects comparison of fear of COVID-19, religiosity, and trust in institutions across countries (Poland, Germany, Slovenia, and Israel) are presented in Figure 2.

The post-hoc analysis with Tukey’s HSD test showed a significant decrease in fear of COVID-19 over time, with a large effect size in each country: Poland (*d* = 1.22, *p* < 0.001), Germany (*d* = 1.08, *p* < 0.001), Slovenia (*d* = 1.01, *p* < 0.001), and Israel (*d* = 1.17, *p* < 0.001). The significantly higher score in Poland compared to Germany (*d* = 1.26, *p* = 0.004) and Slovenia (*d* = 0.30, *p* < 0.001) at T1 was insignificant at T2 (*p* > 0.05). The participants in Israel scored significantly lower compared to all countries at T1: Poland (*d* = −0.70, *p* < 0.001), with a medium effect size, and Germany (*d* = −0.42, *p* < 0.001) and Slovenia (*d* = 0.38, *p* < 0.001), with a small effect size. Furthermore, the participants in Israel also scored significantly lower at T2 compared to all countries: Poland (*d* = −0.55, *p* < 0.001), Germany (*d* = −1.22, *p* < 0.001), and Slovenia (*d* = −0.63, *p* < 0.001), with a medium effect size. There were no significant differences between fear of COVID-19 in Germany and Slovenia at T1 or T2 (*p* > 0.05). Therefore, the differences between countries at T1, showing the highest fear of COVID-19 in Poland and the lowest in Israel, changed over time. During T2, there were no significant differences between Poland, Germany, and Slovenia; however, fear of COVID-19 was significantly lower in Israel compared to all other countries at T2. 

There was no significant interaction between country and time in terms of religiosity, but the post-hoc analysis revealed a significant but very small decrease in religiosity in Germany (*d* = −0.11, *p* = 0.045).

Trust in institutions dropped significantly over time in Germany (*d* = −0.18, *p* < 0.001), although the effect size was very small. There were no other significant differences between T1 and T2 in Poland (*p* > 0.05), Slovenia (*p* > 0.05), and Israel (*p* > 0.05). The participants from Germany scored significantly higher compared to Poland (*d* = 1.01, *p* < 0.001), Slovenia (*d* = 0.97, *p* < 0.001), and Israel (*d* = 0.82, *p* < 0.001), with a large effect size at T1. Similarly, the scores were the highest in Germany at T2 compared to the other countries: Poland (*d* = 0.85, *p* < 0.001), Slovenia (*d* = 0.90, *p* < 0.001), and Israel (*d* = 0.64, *p* < 0.001). However, the difference between Germany and Israel was medium at T2, while it was large at T1. The effect size for the difference between Germany and Poland and Israel was large at both T1 and T2. The details of the interaction between time and country with regard to fear of COVID-19, religiosity, and trust in institutions are presented in Figure 3.

### 3.2. Longitudinal Predictors of Changes in Fear of COVID-19

**Hypothesis** **2** **(H2).** *Religiosity and trust in institutions with regard to country are predictors of change in fear of COVID-19 over a three-month period across Poland, Germany, Slovenia, and Israel*.

The initial step was the correlational analyses, with a Pearson’s *r* coefficient between fear of COVID-19, religiosity, and trust in institutions at T1 and T2. The analysis showed significant but small positive correlations between fear of COVID-19, religiosity, and trust in institutions at T1 and T2. Large positive effects were revealed for fear of COVID-19 at T1 and T2, religiosity at T1 and T2, and trust in institutions between T1 and T2. The details are presented in Figure 4.

H2 was partially confirmed. The GEE analysis was performed for fear of COVID-19 as a dependent variable, country as a factor, and religiosity and trust in institutions as covariates, while the categorical demographic variables considered confounders such as age and gender. The results of the GEE analysis are presented in Table 3. Changes in fear of COVID-19 were significant between the T1 and T2 of the measurement, with a statistically significant decrease in fear of COVID-19 between T1 and T2. These changes were not dependent on the country or age of participants, but female gender, religiosity, and trust in institutions were found to be significant positive longitudinal predictors of fear of COVID-19. Fear of COVID-19 changed relative to changes in religiosity and trust in institutions. The interaction effect between country and religiosity, as well as between country and trust in institutions, was also examined. The findings indicate that no interaction existed between country and religiosity, but a moderating effect of country was observed for the association between trust in institutions and fear of COVID-19. Compared to the Polish participants, those from Israel, Germany, and Slovenia showed less of a regression slope. A significantly stronger association was found between trust in institutions and fear of COVID-19 in the Polish sample, as compared to adults from Germany, Slovenia, and, in particular, Israel (which showed a weaker association). 

## 4. Discussion

In this study, we showed the role of religiosity and trust in institutions in fear of COVID-19 across a three-month period among young adults from Poland, Germany, Slovenia, and Israel. We revealed significant differences in religiosity, trust in institutions, and fear of COVID-19 across the countries. The study also showed religiosity and trust in institutions as positive longitudinal predictors for change in fear of COVID-19 across a three-month period and the moderating role of country. 

Our study showed a significant decrease in fear of COVID-19 and a very small but significant decrease in religiosity and trust in institutions over time. The second measurement of our study was conducted in an improved pandemic situation, with an increase in vaccinated people and a decrease in the mortality rate in each country [50,54]. 

The cross-national comparison showed large differences in religiosity and trust in institutions and small differences in fear of COVID-19. Fear of COVID-19 was highest in Poland and the lowest in Israel. These differences were significant at T1 when the pandemic circumstances were more severe. In the second measurement, during the better pandemic situation (lower number of daily cases and deaths, with a higher number of vaccinations), the differences between Poland, Germany, and Slovenia diminished. However, fear of COVID-19 was the lowest in Israel at T2. Therefore, young adults in Israel scored significantly lower in terms of fear of COVID-19 compared to the other countries, regardless of the pandemic severity. In Israel, certain steps regarding the spread of information about COVID-19 and against vaccination barriers were directed toward specific groups, i.e., young adults and religious minorities [65]. Hence, these actions, at the national level, could have succeed in lowering fear of COVID-19 compared to the other countries. An explanation for the significantly higher fear of COVID-19 in Poland during T1 could be the higher mortality rate compared to the other countries at T1 [50]. Previous research has shown that change in fear of COVID-19 is related to change in mortality rate [51]. 

Religiosity was the highest in Poland compared to other countries, while trust in institutions was the lowest in Poland, similar to Slovenia. In turn, religiosity was the lowest in Slovenia. Israel was characterized by the lowest fear of COVID-19 and religiosity, similar to Germany. Even though Poland and Slovenia represent one cluster of similar values to Catholic Europe [52], the religiosity in our study definitely differed among the young adults. Indeed, even in the Inglehart–Welzel World Cultural Map [52], Poland shares traditional values, while Slovenia, similar to Germany, shares more secular values. Both countries are also classified as post-Soviet countries, which is often a denominator for interpreting similarities and differences compared to Western countries. The common factor in our study turned out to be a lower trust in institutions in those two countries, which might be explained by their similarities in history. On the contrary, the highest trust in institutions was noted in Germany, which also partially carries a post-Soviet history. The analyzed variables in our study significantly differed due to country.

Religiosity was relatively stable over time in Poland, Slovenia, and Israel. However, we found a significant but small decrease in Germany. Previous research in European countries has shown that trust in institutions is related to a lower mortality rate [42]. However, our study showed that trust in institutions declined over time, even though the mortality rate decreased.

We found positive but small relationships between fear of COVID-19, religiosity, and trust in institutions in the total sample. At both study timepoints, religiosity was positively related to trust in institutions. Therefore, higher religiosity was related to higher trust in institutions. We assume that the small effect size could be due to the diversity of identification with religion in our study (11 categories), as a previous study showed that this relationship depends on a specific religion and cultural differences [44,45,46,47]. Nevertheless, unlike other research [44,45,47], we confirmed significant positive relationships in a cross-national study. We assume that religious people respect rules as much as those who trust in institutions. Furthermore, the stimulation of the social cohesion and cooperation attitude through collective rituals as a manifestation of religious life [66] may explain this positive relation as well. 

Finally, we showed that changes in religiosity and trust in institutions are positive predictors of changes in fear of COVID. The changes in fear of COVID-19 were dependent on female gender but independent of country and age. Our results regarding the role of gender are in line with previous research on fear of COVID-19 [49,50,51,52]. Furthermore, we showed that country does not play a moderating role between religiosity and fear of COVID. 

Our findings contrast with general negative relationships between religiosity and fear and anxiety [10], and have a positive relationship with mental health from a longitudinal perspective [31,32]. Our study showed that religiosity does not play a buffer role in the decline in fear of COVID-19, as was revealed in previous papers regarding mental health problems [11,12,13]. It could be argued that higher religiosity is related to an increase in fear of COVID-19 due to the negative attitude of the Catholic church toward vaccinations for COVID-19 [67]. The lack of an ethically accepted remedy for the disease could increase the fear of the disease in religious people. This relationship was noted in the total sample, which was composed of highly secularized countries, such as Slovenia and Germany, and highly religious nations such as Poland. Therefore, religiosity is a longitudinal predictor of fear of COVID-19 regardless of country. 

Furthermore, we showed that trust in institutions is a positive longitudinal predictor of fear of COVID-19 and revealed the moderating role of country in the positive relationship between trust in institutions and fear of COVID-19. The strongest relationship was observed in Poland compared to all other countries. The metanalysis on the global prevalence of mental health during the pandemic showed that the prevalence of mental health issues differs across countries and depends on countries’ preparedness to respond, health policies, and economic vulnerabilities [3]. It was revealed that the institutions in all countries, but particularly in Poland, failed to comfort their citizens during the COVID-19 pandemic, as trust in institutions elevated the change in fear of COVID-19 over time.

### Limitations

A strong point of the study is the cross-national longitudinal design among representative samples. However, there are several limitations to this research. The first is that all the introduced measurements were based on self-assessment. Furthermore, even though the measurement of religiosity is widely used, this is a one-item measurement. Moreover, the study design included change across a short three-month period. Therefore, more measurement timepoints would allow for the evaluation of a trend. 

## 5. Conclusions

Our study filled the gap regarding the role of religiosity and trust in institutions across Poland, Germany, Slovenia, and Israel in fear of COVID-19 among young adults. 

We showed that religiosity and trust in institutions not only fail to buffer the fear of COVID-19, but actually enhance it. The main conclusion is that both governmental and religious institutions should place greater emphasis on health policy communication during unexpected and difficult situations, so as not to enhance mental health problems.

Furthermore, a cross-cultural perspective showed that the results differed across countries. Particularly, Polish governmental institutions should draw conclusions for further activity to increase believers’ and citizens’ trust in their judgment. 

## Figures and Tables

**Table 1:** Demographic characteristics of the study sample.

Demographic Variables	Total		Poland		Germany		Slovenia		Israel	
	*n*	%	*n*	%	*n*	%	*n*	%	*n*	%
Gender										
Women	935	54.30	222	49.80	224	53.60	247	57.30	242	56.50
Men	782	45.40	221	49.60	193	46.20	183	42.50	185	43.20
Did not want to say	6	0.30	3	0.70	1	0.20	1	0.20	1	0.20
Age										
20–30 years	840	48.80	236	52.90	202	48.30	202	46.90	200	46.70
31–40 years	883	51.20	210	47.10	216	51.70	229	53.10	228	53.30
Place of residence										
Village	426	24.70	155	34.80	71	17.00	162	37.60	28	8.90
Town (under 20,000 inhabitants)	310	18.00	63	14.10	84	20.10	120	27.80	43	10.00
City (20,000–99,000 inhabitants)	368	21.40	82	18.40	98	23.40	62	14.40	126	29.40
City (100,000–500,000 inhabitants)	380	22.10	85	19.10	82	19.60	65	15.10	148	34.60
Agglomeration (over 500,000 inhabitants)	239	19.90	61	13.70	83	19.90	22	5.10	73	17.10
Employment status										
Employed	1227	71.20	324	72.60	304	72.70	284	65.90	315	73.60
Unemployed	399	23.20	93	20.90	91	21.80	123	28.50	92	21.50
Self-employed	97	5.60	29	6.50	23	5.50	24	5.60	21	4.90
Religion										
No religion	523	30.40	97	21.70	185	44.30	209	48.50	32	7.50
Buddhist	27	1.60	4	0.90	15	3.60	4	0.90	4	0.90
Catholic	602	34.90	328	73.50	104	24.90	169	39.20	1	0.20
Hindu	2	0.10	0	0.00	2	0.50	0	0.00	0	0.00
Jehovah’s Witness	11	0.60	5	1.10	3	0.70	1	0.20	2	0.50
Jewish	379	22.00	0	0.00	1	0.20	2	0.50	376	87.90
Methodist	1	0.10	1	0.20	0	0.00	0	0.00	0	0.00
Muslim	44	2.60	2	0.40	22	5.30	12	2.80	8	1.90
Orthodox (Eastern, Russian, Greek)	51	3.00	5	1.10	33	7.90	12	2.80	1	0.20
Protestant	46	2.70	1	0.20	39	9.30	5	1.20	1	0.20
Other	37	2.10	3	0.70	14	3.30	17	3.90	3	0.70
Total	1723	100	446	25.90	418	24.30	431	25.00	428	24.80

**Table 2:** Repeated-measures two-way ANOVA statistics for fear of COVID-19, religiosity, and trust in institutions over time and across countries among young adults from Germany, Israel, Poland, and Slovenia.

	Fear of COVID-19				Religiosity				Trust in Institutions			
Effect	*F*	*df*	*p*	η²_p_	*F*	*df*	*p*	η²_p_	*F*	*df*	*p*	η²_p_
Time	3077.54	1, 1719	<0.001	0.64	5.50	1, 1719	0.019	0.003	15.78	1, 1719	<0.001	0.01
Country	40.90	3, 1719	<0.001	0.07	44.61	3, 1719	<0.001	0.07	97.87	3, 1719	<0.001	0.15
Time × Country	5.72	3, 1719	<0.001	0.01	1.85	3, 1719	0.136	0.003	4.64	3, 1719	0.003	0.01

Note: ANOVA, analysis of variance; time 1, the first measurement in February 2021; time 2, the second measurement in May–June 2021.

**Table 3:** Longitudinal predictors of change in fear of COVID-19 between T1 and T2 among young adults (*N* = 1723).

Parameter	*B*	SE *B*	95% CI		Wald’s Statistics	
			LL	UL	*χ*^2^ (1)	*p*
Constant	12.80	0.83	11.16	14.43	235.72	<0.001
Time T2 (vs. T1)	−7.86	0.14	−8.12	−7.59	3324.41	<0.001
**Country (vs. Poland)**						
Israel	1.69	1.03	−0.33	3.70	2.70	0.100
Slovenia	−0.62	1.06	−2.70	1.45	0.35	0.556
Germany	−0.39	1.20	−2.74	1.97	0.10	0.747
Female gender (vs. male)	0.87	0.28	0.32	1.41	9.64	0.002
Younger adults (vs. older)	−0.47	0.28	−1.01	0.07	2.93	0.087
Religiosity	0.73	0.31	0.11	1.34	5.38	0.020
Trust in institutions	0.28	0.04	0.20	0.36	50.72	<0.001
**Country (vs. Poland)** × **religiosity**						
Israel × religiosity	−0.71	0.38	−1.46	0.03	3.54	0.060
Slovenia × religiosity	0.21	0.46	−0.68	1.10	0.21	0.644
Germany × religiosity	0.50	0.43	−0.34	1.34	1.35	0.246
**Country (vs. Poland)** × **trust in institutions**						
Israel × trust in institutions	−0.24	0.06	−0.35	−0.13	17.78	<0.001
Slovenia × trust in institutions	−0.15	0.06	−0.28	−0.03	6.11	0.013
Germany × trust in institutions	−0.19	0.06	−0.30	−0.08	11.88	0.001

Note: CI, confidence interval; LL, lower level of the confidence interval; UL, upper level of the confidence interval; T1, the first measurement; T2, the second measurement; younger adults, aged 20–30 years; older adults, aged 31–40 years.

## Data Availability

This study forms part of the international research project “Mental health of young adults during the COVID-19 pandemic in Poland, Germany, Slovenia, and Israel: A longitudinal study” [56], registered at the Center for Open Science (OSF). The datasets used and analyzed during the current study are available from the corresponding author upon reasonable request.
